# Supplementary material for: Temperature-dependence of early development of zebrafish and the consequences for laboratory use and animal welfare
Source: PLoS One. 2025 Dec 31;20(12):e0340193. doi: 10.1371/journal.pone.0340193 (PMC12755749; doi:10.1371/journal.pone.0340193)
Supplement: S1 Table — (PDF) [file pone.0340193.s001.pdf]

Table S1: Descriptions of the embryonic stages determined during the high-resolution time series.

Stage	Abbreviation	Description
4 cell	4c	2 x 2 array of blastomeres
8 cell	8c	2 x 4 array of blastomeres
16 cell	16c	4 x 4 array of blastomeres
32 cell	32c	2 regular tier (horizontal rows) of blastomeres, 4 x 8 array
64 cell	64	3 regular tier of blastomeres
128 cell	128	5 blastomeres tier, cleavage planes irregular
256 cell	256c	7 - 9 irregular blastomeres tiers, yolk syncytial layer develops
oblong	O	> = 11 irregular blastomeres tiers, beginning of blastodisc cell asynchrony and flattening. Flattening produces an elliptical shape. Animal-vegetal axis of the blastula shortens, with the blastodisc compressing down upon the yolk cell
sphere	S	Continued shortening along tha animal-vegetal axis generates a late blastula of smooth and approximately spherical shape, flat border between blastodisc and yolk, start of yolk cell bulging (doming) toward animal pole as epiboly begins
epiboly	E	Epiboly is the thinning and spreading of the yolk syncytical layer and the blastodisc over the yolk cell. 30%-50% epiboly.
shield	SH	germ ring/ shield visible from animal pole. After reaching a 50% epiboly a thickened marginal region (germ ring), nearly simultaneously all around the blastoderm rim. Convergence movements then, nearly as rapidly, produce a local accumulation of cells at one position along the germ ring, the embryonic shield
75 epiboly	75E	Dorsal side distinctly thicker, thin evacuation zone on ventral side
90 epiboly	90E	Brain rudiment thickened, notochord rudiment distinct from segmental plate
bud	B	tail bud prominent, early polster, along dorsal side the neural plate is thickened along the embryonic axis. The thickening is most prominent near the animal pole in the prospective head region, where the head will form
3 somite	3s	first three somites furrow
6 somite	6s	brain primordium has distinctively thickened, eye primordium
8 somite	8s	8 - 10 somites without otic placode
10 somite	10s	otic placode developed
14 somite	14s	yolk cell begins to invert and look like kidney-bean. Somites begin to take on chevron shape
18 somite	18s	yolk cell extension is clearly delimited from the yolk ball as the tail straightens out
21 somite	21s	lens primordium
26 somite	26s	Straightening of posterior trunk is nearly completed, but elongating tail still curves ventrally. Cerebellar primordium is prominent
prim	P	Cerebellum is evident at the hindbrain/midbrain boundary region

Eye pigmentation	EP	eye pigmentation begins
Body pigmentation	BP	body pigmentation begins

Stages were categorized according to the developmental criteria of Kimmel et al. (1995). The table shows the selection of criteria from Kimmel et al. (1995) used to determine the respective stage.
