# Supplementary material for: Temperature-dependence of early development of zebrafish and the consequences for laboratory use and animal welfare
Source: PLoS One. 2025 Dec 31;20(12):e0340193. doi: 10.1371/journal.pone.0340193 (PMC12755749; doi:10.1371/journal.pone.0340193)
Supplement: S2 Table — (PDF) [file pone.0340193.s002.pdf]

Table S2: Delays in development observed for different endpoints computed with confidence interval separately for each replicate.

		Hours of delay		
**Endpoint**	dpf	min	average	max
**Early Developmental Stages (Body Pigmentation (BP)*)**	1			
R1		3	4	4.5
R2		1	3	5
Coefficient of Variation		0.71	0.20	0.07
**Onset of Heartbeat**	2			
R1		3	3	5
R2		4	5.5	6
Coefficient of Variation		0.20	0.42	0.13
**Hatching**	3			
R1		3.5	5	11.5
R2		1	6	14
Coefficient of Variation		0.79	0.13	0.14
**Body Length hr**	5			
R1		-6	3.4	10
R2		17.8	22	25.6
Coefficient of Variation		2.85	1.04	0.62
**Body Length lr**	5			
R1		4.6	12.9	19.1
R2		5	10.7	15.6
R3		-0.8	12.3	18.8
Coefficient of Variation		1.10	0.10	0.11
**Eye Size**	5			
R1		-1.5	8.26	19.5
R2		7.1	17.9	28.1
R3		6.6	16.7	27.4
Coefficient of Variation		1.19	0.37	0.19
**Yolk Sac Consumption**	5			
R1		12.4	22.4	31.3
R2		6.8	13.2	18.5
R3		7	16.8	25.6
Coefficient of Variation		0.36	0.27	0.26

The coefficient of variation between replicates is given for all endpoints.

*the final stage observed in the early developmental period and its associated temporal endpoint.
hr = high-resolution time series, lr = low-resolution time series
