# Supplementary material for: Temperature-dependence of early development of zebrafish and the consequences for laboratory use and animal welfare
Source: PLoS One. 2025 Dec 31;20(12):e0340193. doi: 10.1371/journal.pone.0340193 (PMC12755749; doi:10.1371/journal.pone.0340193)
Supplement: S1 Fig — (PDF) [file pone.0340193.s003.pdf]

Body length

High-resolution 26 °C

	Estimate	Std. Error	t value	Pr(>|t|)
(Intercept)	2.95774	0.03321	89.06	<2e-16 ***
Approximate significance of smooth terms:				
	edf	Ref.df	F	p-value
s(hpf)	3	3.001	1486.5	<2e-16 ***
s(replicate)	0.992	1	358.7	<2e-16 ***
R-sq.(adj) = 0.7935 Deviance explained = 79.4%				
GCV score = 0.011127 Scale est. = 0.011083 n = 1262				

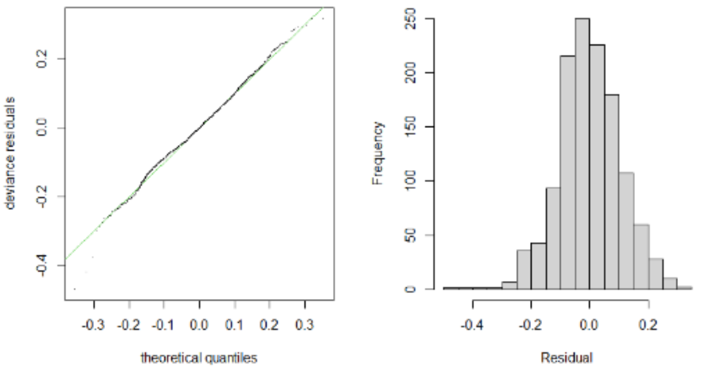

High-resolution 28 °C

	Estimate	Std. Error	t value	Pr(>|t|)
(Intercept)	3.03041	0.02754	110	<2e-16 ***
Approximate significance of smooth terms:				
	edf	Ref.df	F	p-value
s(hpf)	3.0294	3.056	3463.69	<2e-16 ***
s(replicate)	0.9935	1	44.71	3.06e-11 ***
R-sq.(adj) = 0.8644 Deviance explained = 86.5%				
GCV score = 0.0082589 Scale est. = 0.0082344 n = 1692				

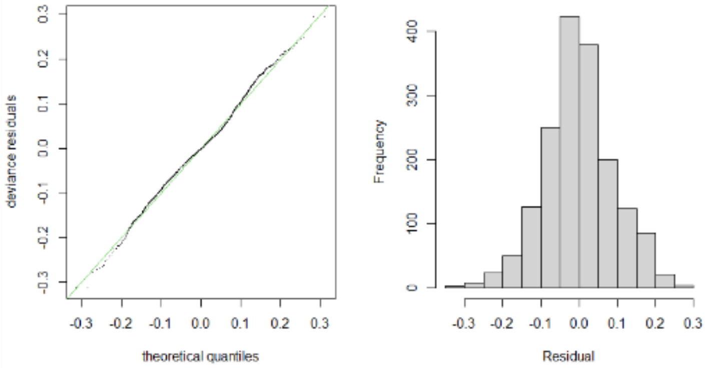

Low-resolution 26 °C

	Estimate	Std. Error	t value	Pr(>|t|)
(Intercept)	3.54389	0.01541	229.9	<2e-16 ***
Approximate significance of smooth terms:				
	edf	Ref.df	F	p-value
s(hpf)	2	2	477.57	<2e-16 ***
s(replicate)	1.561	2	5.102	0.00551 **
R-sq.(adj) = 0.8491 Deviance explained = 85.2%				
GCV score = 0.0090238 Scale est. = 0.0087845 n = 172				

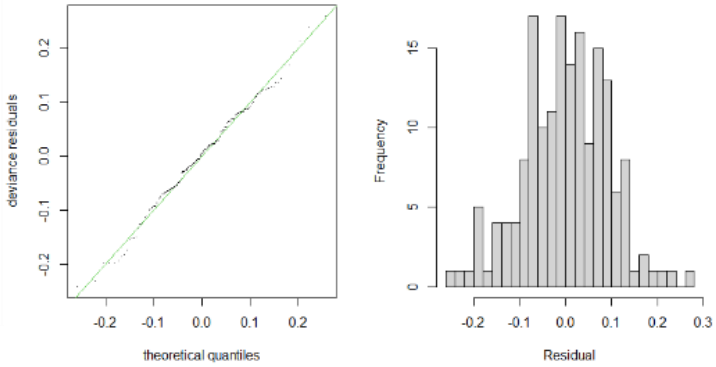

Low-resolution 28 °C

	Estimate	Std. Error	t value	Pr(>|t|)
(Intercept)	3.65587	0.01612	226.8	<2e-16 ***
Approximate significance of smooth terms:				
	edf	Ref.df	F	p-value
s(hpf)	2	2	505.038	<2e-16 ***
s(replicate)	1.624	2	5.158	0.0056 **
R-sq.(adj) = 0.8577 Deviance explained = 86.1%				
GCV score = 0.008534 Scale est. = 0.0083019 n = 170				

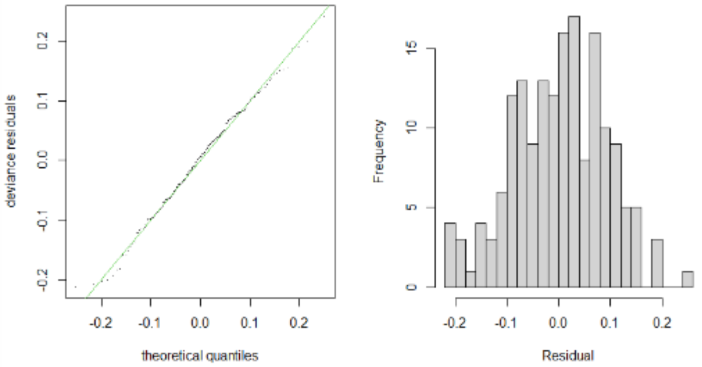

Fig. S1: Parameters and diagnostic plots for non-parametric Shape Constrained Additive Model (SCAM with integrated smoothness) with a random effects term for replicate.
