# Supplementary material for: Temperature-dependence of early development of zebrafish and the consequences for laboratory use and animal welfare
Source: PLoS One. 2025 Dec 31;20(12):e0340193. doi: 10.1371/journal.pone.0340193 (PMC12755749; doi:10.1371/journal.pone.0340193)
Supplement: S2 Fig — (PDF) [file pone.0340193.s004.pdf]

	Estimate	Std.Error	t value	Pr(>|t|)
(Intercept)	-15.7718	1.358748	-11.61	<2e-16 ***
temperature	1.404162	0.101353	13.85	<2e-16 ***
I(temperature^2)	-0.02507	0.001878	-13.35	<2e-16 ***

Residual standard error: 0.1206 on 256 degrees of freedom

Multiple R-squared: 0.6277, Adjusted R-squared: 0.6248

F-statistic: 215.8 on 2 and 256 DF, p-value: < 2.2e-16

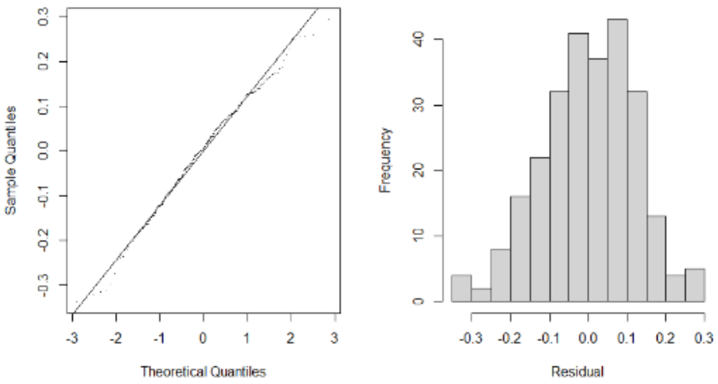

Fig. S2: Model diagnostics for the regression of body length over temperature with a quadratic model.
