# Supplementary material for: Temperature-dependence of early development of zebrafish and the consequences for laboratory use and animal welfare
Source: PLoS One. 2025 Dec 31;20(12):e0340193. doi: 10.1371/journal.pone.0340193 (PMC12755749; doi:10.1371/journal.pone.0340193)
Supplement: S3 Fig — (PDF) [file pone.0340193.s005.pdf]

Yolk sac

Low-resolution 26 °C

Parametric coefficients:				
	Estimate	Std. Error	t value	Pr(>|t|)
(Intercept)	0.282512	0.004489	62.94	<2e-16 ***
Approximate significance of smooth terms:				
	edf	Ref.df	F	p-value
s(hpf)	2	2	119.419	< 2e-16 ***
s(replicate	1.674	2	9.375	0.000107 ***
R-sq.(adj) = 0.5914 Deviance explained = 60%				
GCV score = 0.00059032 Scale est. = 0.00057464 n = 176				

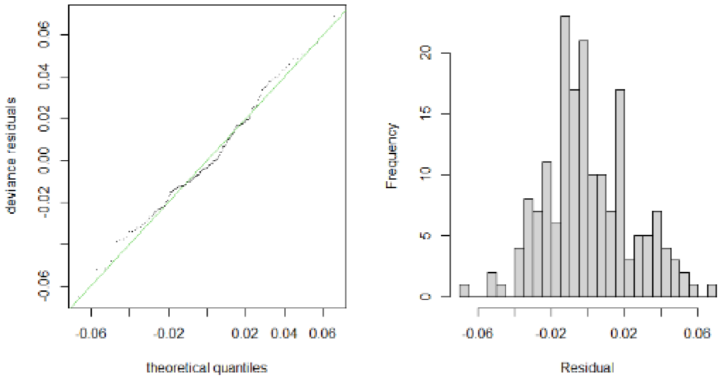

Low-resolution 28 °C

Parametric coefficients:				
	Estimate	Std. Error	t value	Pr(>|t|)
(Intercept)	0.261565	0.002007	130.3	<2e-16 ***
Approximate significance of smooth terms:				
	edf	Ref.df	F	p-value
s(hpf)	2	2	155.358	< 2e-16 ***
s(replicate	0.0133	2	0.006	0.649
R-sq.(adj) = 0.6409 Deviance explained = 64.5%				
GCV score = 0.00070851 Scale est. = 0.00069624 n = 174				

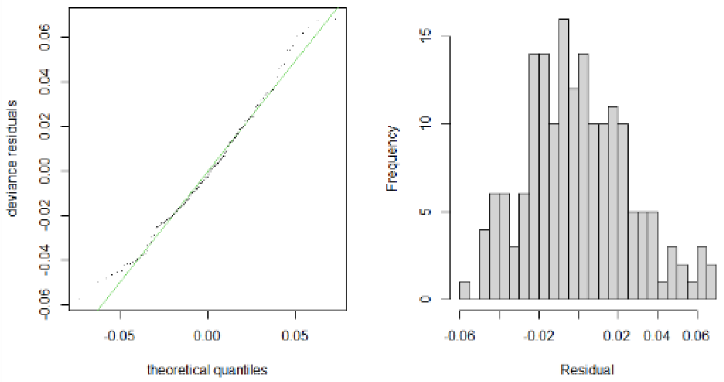

Eye size

Low-resolution 26 °C

	Estimate	Std. Error	t value	Pr(>|t|)
(Intercept)	0.06399	0.00252	25.39	<2e-16 ***
Approximate significance of smooth terms:				
	edf	Ref.df	F	p-value
s(hpf)	2	2	140.98	< 2e-16 ***
s(replicate)	1.922	2	20.14	1.16e-08 ***
R-sq.(adj) = 0.6413 Deviance explained = 64.9%				
GCV score = 4.4e-05 Scale est. = 4.2769e-05 n = 176				

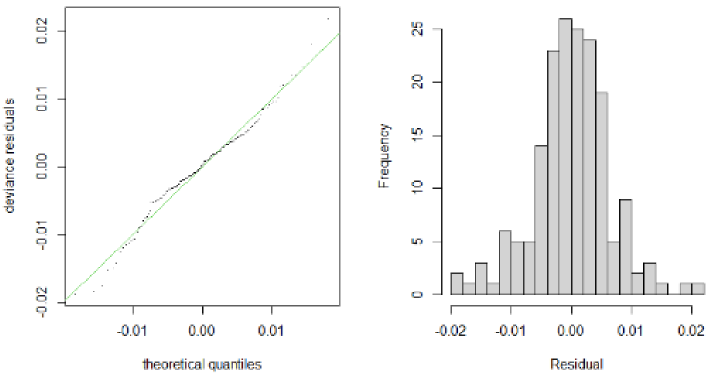

Low-resolution 28 °C

	Estimate	Std. Error	t value	Pr(>|t|)
(Intercept)	0.069391	0.001438	48.27	<2e-16 ***
Approximate significance of smooth terms:				
	edf	Ref.df	F	p-value
s(hpf)	2	2	129.82	< 2e-16 ***
s(replicate)	1.683	2	11.21	2.02e-05 ***
R-sq.(adj) = 0.6166 Deviance explained = 62.5%				
GCV score = 5.8763e-05 Scale est. = 5.719e-05 n = 175				

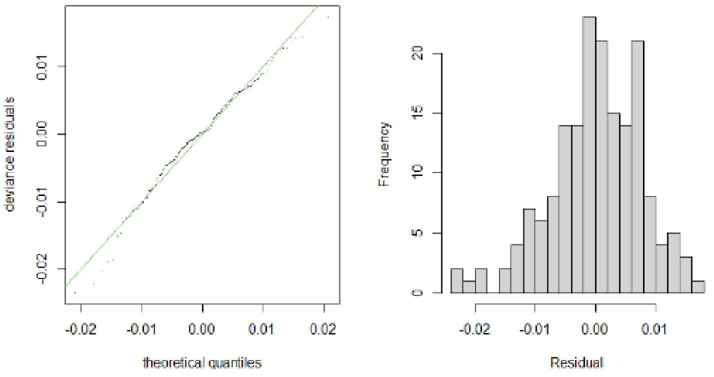

Fig. S3: Parameters and diagnostic plots for non-parametric Shape Constrained Additive Model (SCAM with integrated smoothness) with a random effects term for replicate.
