# Supplementary material for: Temperature-dependence of early development of zebrafish and the consequences for laboratory use and animal welfare
Source: PLoS One. 2025 Dec 31;20(12):e0340193. doi: 10.1371/journal.pone.0340193 (PMC12755749; doi:10.1371/journal.pone.0340193)
Supplement: S3 Table — (PDF) [file pone.0340193.s006.pdf]

Table S3: Representative overview of temperature-dependent developmental differences in zebrafish embryos at 26°C and 28°C at three early stages: 14, 18, and 24 hpf.

	**14 hpf**	**18 hpf**	**24 hpf**
**26 °C**	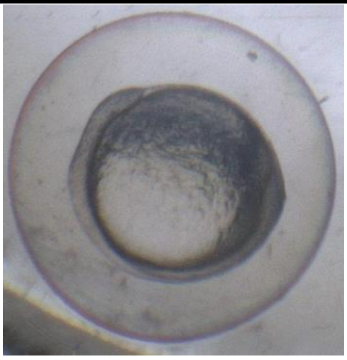 *Bud*	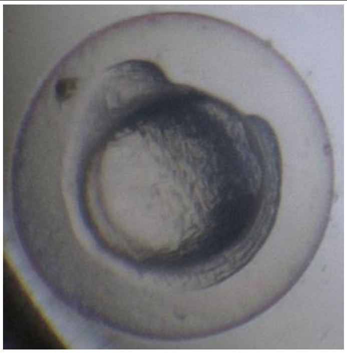 *8 somite*	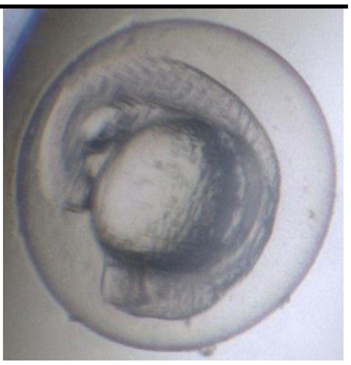 *21 somite*
**28 °C**	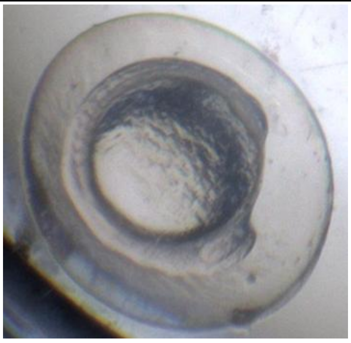 *8 somite*	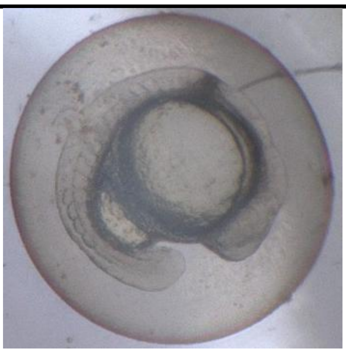 *18 somite*	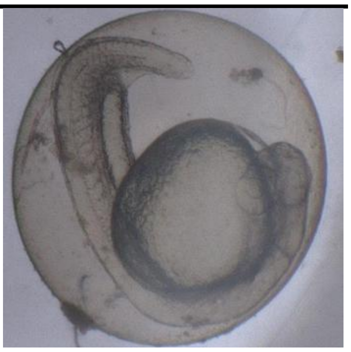 *prim*

The table highlights morphological differences observed at each time point, illustrating the influence of incubation temperature on embryonic development.
