# Supplementary material for: Temperature-dependence of early development of zebrafish and the consequences for laboratory use and animal welfare
Source: PLoS One. 2025 Dec 31;20(12):e0340193. doi: 10.1371/journal.pone.0340193 (PMC12755749; doi:10.1371/journal.pone.0340193)
Supplement: S4 Table — (PDF) [file pone.0340193.s007.pdf]

Table S4: Delay in hours post fertilization for zebrafish embryos to reach developmental stages at 26°C and 28°C.

Stage	Abbreviation	n (26 °C)	n (28 °C)	Difference in medians / h	CI	p-value
4-cell	4c	29	20	0.0	0.0 – 0.0	-
8-cell	8c	20	28	0.0	0.0 – 0.0	-
16-cell	16c	8	6	0.0	0.0 – 0.0	-
32-cell	32c	20	34	0.0	0.0 – 0.0	-
64-cell	64c	14	14	0.0	0.0 – 0.0	-
128-cell	128c	21	12	0.0	0.0 – 0.0	-
256-cell	256c	24	33	0.5	0.0 – 0.0	-
oblong	O	22	26	0.0	0.0 – 0.0	-
sphere	S	17	18	1.0	0.0 – 0.0	-
epiboly	E	28	33	0.0	0.0 – 1.5	0.157
shield	SH	40	41	1.0	0.25 – 1.5	< 0.0001
75-epiboly	75E	29	26	1.0	1.5 – 2.5	< 0.0001
90-epiboly	90E	41	44	2.0	0.25 – 2.0	< 0.0001
bud	B	43	42	2.0	2.5 – 3.0	< 0.0001
3-somite	3s	38	37	2.0	2.0 – 3	< 0.0001
6-somite	6s	34	44	2.0	2.5 – 3.5	< 0.0001
8-somite	8s	36	25	3.0	2.25 – 3.5	< 0.0001
10-somite	10s	31	31	2.0	1.5 – 3.5	< 0.0001
14-somite	14s	24	13	2.5	1.5 – 3.0	< 0.0001
18-somite	18s	36	36	3.0	2.5 – 4.0	< 0.0001
21-somite	21s	43	41	3.0	2.75 – 4.0	< 0.0001
26-somite	26s	37	43	4.0	3.0 – 5.25	< 0.0001
prim	P	43	39	5.0	4.25 – 6.25	< 0.0001
pigmented eyes	EP	37	41	4.0	4.5 – 6.0	< 0.0001
pigmented body	BP	44	46	3.0	3.25 – 5.0	< 0.0001

n = number of embryos this stage was observed in, CI = bootstrapped confidence interval of difference in medians confined by replicate, p-value: derived from a permutation test with replicate as blocking factor (2000 resamples) and corrected for alpha inflation with the Bonferroni Method.
