# Supplementary material for: Temperature-dependence of early development of zebrafish and the consequences for laboratory use and animal welfare
Source: PLoS One. 2025 Dec 31;20(12):e0340193. doi: 10.1371/journal.pone.0340193 (PMC12755749; doi:10.1371/journal.pone.0340193)
Supplement: S5 Fig — (PDF) [file pone.0340193.s009.pdf]

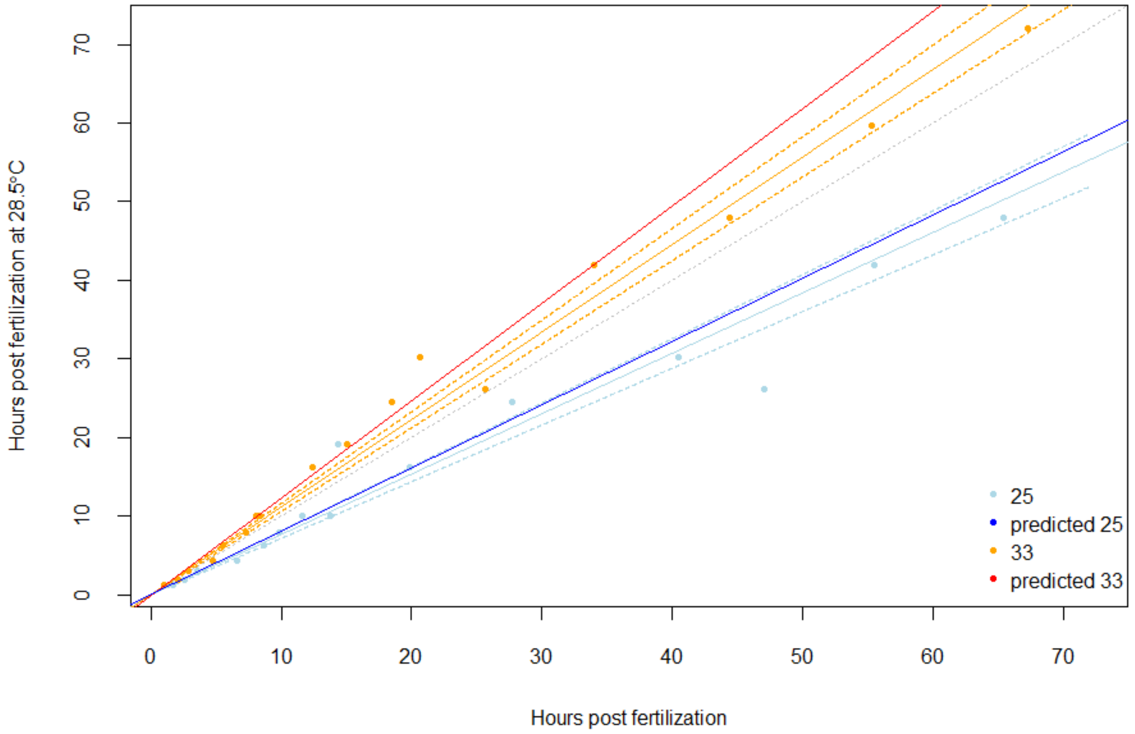

Fig. S5: Relationship between the hours post fertilization (hpf) at different temperatures and the corresponding normalized hpf at 28.5°C, comparing observed data points with predicted values based on fitted regression lines (adapted from Kimmel et al. (1995)). Raw data is listed in Table S5.
