# Supplementary material for: Temperature-dependence of early development of zebrafish and the consequences for laboratory use and animal welfare
Source: PLoS One. 2025 Dec 31;20(12):e0340193. doi: 10.1371/journal.pone.0340193 (PMC12755749; doi:10.1371/journal.pone.0340193)
Supplement: S5 Table — (PDF) [file pone.0340193.s010.pdf]

Table S5: Raw data of developmental rates derived from Kimmel et al. (1995) by using Web Plot Digitizer (Raharzi, 2024).

hpf at 25 °C	hpf at 28.5 °C	hpf at 33 ° C
1.7	1.3	1
2.6	2	2.1
3.6	3	2.9
6.6	4.3	4.7
8.7	6.3	5.5
9.8	8	7.3
11.6	10	8.1
13.8	10.1	8.4
14.4	16.3	12.4
19.9	19.2	15.0
27.7	24.5	18.5
40.5	26.1	20.6
47.0	30.2	25.6
55.5	41.9	34.0
65.4	47.9	44.4
76.7	59.7	55.3
87.9	72.1	67.3

Shown are the hpf at 28.5°C, which is the standard incubation temperature, and the development of the eleutheroembryos in hpf when incubated at higher or lower temperatures.
