# Supplementary material for: Temperature-dependence of early development of zebrafish and the consequences for laboratory use and animal welfare
Source: PLoS One. 2025 Dec 31;20(12):e0340193. doi: 10.1371/journal.pone.0340193 (PMC12755749; doi:10.1371/journal.pone.0340193)
Supplement: Attachment — Submitted filename: PlosOne_Answer-to-Reviewers.docx [file pone.0340193.s012.docx]

**Author answers to Review comments -** Temperature-dependence of Early Development of Zebrafish and the Consequences for Laboratory Use and Animal Welfare

| **Line** | **Comment – Reviewer 1** | **Answer** |
| --- | --- | --- |
| *325-328* | This study yielded very interesting results for the scientific community, providing new data on the influence of temperature on the development of zebrafish embryos by comparing different temperature regimes (24, 26, 28 and 30 °C) or after exposure to temperature variations (in the case of embryos in the high-resolution time series exposed to frequent removal from the incubator l.325-328). The exploration of this last point might improve the manuscript by exposing the fish to fluctuating or oscillating temperature regimes in order to describe the influence of these variations more precisely. | We agree that exploring the effects of fluctuating temperature could provide valuable insights into the influence of temperature variability on zebrafish development. Nevertheless, the study did not aim to assess the role of fluctuating temperatures. In fact, we ended up analyzing these fluctuations because of the nature of the experiment required the frequent removal from the incubator, we wanted to be transparent about.  Additional exploration of influence of fluctuations would not directly address our primary research objective, which focused on defining temperature-dependent developmental delays during early embryogenesis. Primary conclusions about delays were therefore drawn from low-resolution time series results. |
| 179  247-255 | high temporal resolution microscopy described for the study of early stages of development (between 1 hpf and 40 hpf) can induce phototoxicity with adverse effects on living samples, consequences of illumination that can affect developmental dynamics, sample morphology and reproducibility of results. This should be discussed on the same basis as repeated handling of the embryos (l.179), genetic or conditions of incubation (l.247-255). | We have considered the potential negative effects of microscope light on body length development in l.338-345. |
| 290-293 | Malformation and death have been associated at temperature above 32.5°C (l.290-293). Did the authors observe higher mortality rates at 30°C in their experiments? Did any malformations occur? If so, can it be commented on ? | We have included additional information on the mortality and malformation rate at 24 and 30°C in l.295-297, emphasizing that they are elevated. |
| 294-295 | In paragraph 3.1.2, there are discrepancies between the text and the figures (l.294-295. This should be corrected. | We have corrected the timing of heartbeat onset, and it now corresponds accurately with the values shown in Fig. 2 (l.302). |
| 319-320 | In paragraph 3.1.4, the authors showed that the body length of the embryos was smaller when comparing the high and low resolution time series (l.319-320). Adding a short sentence at this level on the batch variation before returning to this point at the end of the paragraph could make the table easier to read. Please also comment the potential phototoxicity effect. | To clarify the context of handling effects between the high- and low-resolution time series, we have rearranged the section on batch variation to directly follow the discussion of discrepancies between experiments. We have also expanded this section to include a comment on potential phototoxicity effects (l. 338-355). |
|  | In paragraph 3.2.1, the authors monitored yolk sac consumption 72, 96 and 119 hours post fertilization. Would it be possible to reinforce these results by providing information on the opening of the mouth and anus and/or the development of the intestine? | Unfortunately, the opening of the mouth and anus and the intestine development is very hard to determine from our pictures, because of light settings and zoom, as this was not planned to be analyzed at the moment of acquisition. |

| **Line** | **Comment – Reviewer 2** | **Answer** |
| --- | --- | --- |
| 114 | how was anaesthesia achieved? | We have incorporated detailed information regarding the anaesthesia procedure into the manuscript (l.118-119). |
| 116 | was a fixative used or an immobilizer? Which solution was used? | We have added information on the fixative solution to the manuscript (l.122) |
| 121 | if 53-61 individuals were used from a total of 72, what happened to the remaining individuals? | The numbers reported correspond to the larvae that were successfully fixed. Because we strictly required proper eye overlap, some larvae did not meet this criterion and were therefore excluded from the analysis (as indicated in l. 126: “successfully fixated”). |
| 121 | fixated” or immobilized? | Because the larvae were first immobilized in benzocaine-enriched water and subsequently fixated in gelatine, we used the term “fixated” (l. 126). |
| 296 | this difference should be included in the figure to better visualize the statistical differences between temperatures. The same applies to the hatching results | We adapted the figures to include the length and direction of delay by means of an arrow (compare Fig.2 and Fig.3). |
